# A Method for Ventricular Late Potentials Detection Using Time-Frequency Representation and Wavelet Denoising

**DOI:** 10.5402/2012/258769

**Published:** 2012-08-26

**Authors:** Matteo Gadaleta, Agostino Giorgio

**Affiliations:** Dipartimento di Elettrotecnica ed Elettronica, Politecnico di Bari, Via E. Orabona, 4 70125 Bari, Italy

## Abstract

This study proposes a method for ventricular late potentials (VLPs) detection using time-frequency representation and wavelet denoising in high-resolution electrocardiography (HRECG). The analysis is performed both with the signal averaged electrocardiography (SAECG) and in real time. A comparison between the temporal and the time-frequency analysis is also reported. In the first analysis the standard parameters QRSd, LAS40, and RMS40 were used; in the second normalized energy in time-frequency domain was calculated. The algorithm was tested adding artificial VLPs to real ECGs.

## 1. Introduction

**Figure 1:**
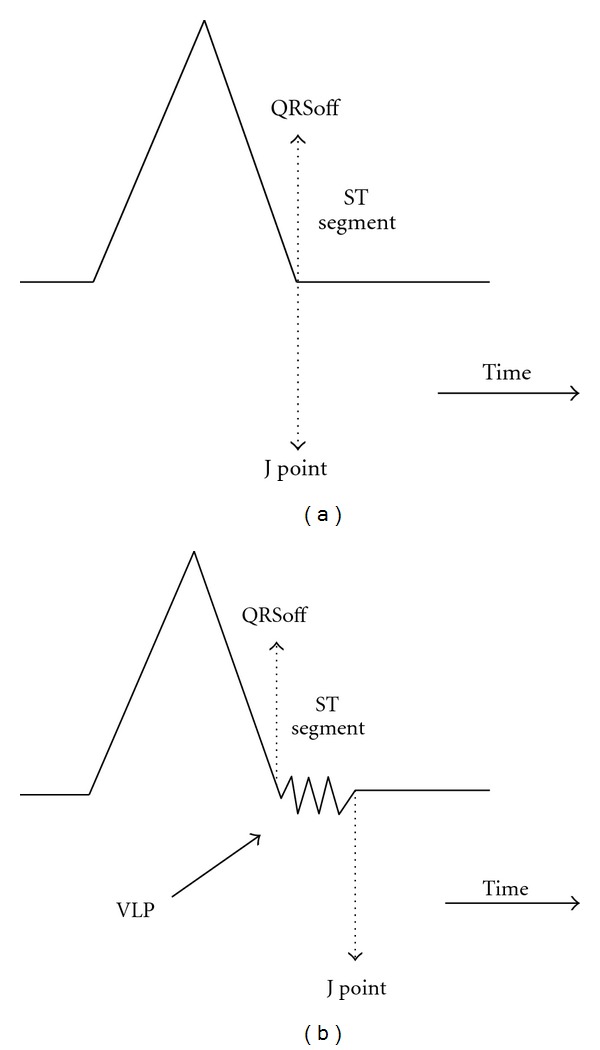
Schematic ECG without (a) and with very exalted VLPs (b). Some characteristic points are also marked.

The ventricular late potentials (VLP) are high-frequency (in relation to the bandwidth of the electrocardiographic signal) and very-low-intensity signals. The presence of VLPs in the electrocardiographic signal has been associated with damages in the ventricular myocardial tissues. The necrosis or ischemic death of myocardial cells causes the formation of high-resistivity areas, where the propagation of cardiac action potential is delayed. This phenomenon affects the electrocardiographic signal with the presence of electrical activity, although of low intensity, between the end of the QRS complex and the initial part of the ST segment, where it should not be (Figure 1) [1]. 

VLPs are localized at the end of QRS complex and in the initial part of the ST segment. Their intensity is at least two orders of magnitude smaller than the electrocardiographic signal, so they are usually “hidden” below the noise produced by the acquisition hardware and the electrical activity not related to the heart. For these reasons VLPs are not easily visible on the ECG.

Several statistical studies demonstrated a correlation between the presence of VLPs and the possibility of sudden cardiac death due to arrhythmia, often tachycardia. Patients with previous ischemic events are the most at risk. A correct VLPs detection makes the prevention of this serious malignant arrhythmias possible.

A classic electrocardiographic signal has amplitude of the order of a few mV and it contains most of the information at frequencies below 100 Hz. VLPs, if present, are considered nonstationary and non-Gaussian signals with an amplitude between 1 and 20 *μ*V.

Table 1 summarizes the main features of the VLP.

The low-amplitude and high-frequency dispersion makes VLPs detection very difficult, often the signal is dominated by the noise. It is therefore necessary to process the signal to drastically reduce the noise level.

In this paper we propose a method to detect VLPs in two different conditions: analyzing a few minutes of prerecorded ECG with the use of signal averaged electrocardiography (SAECG) and examining the ECG during its acquisition. The first analysis allows the identification of weaker VLPs, while the second can be used for real-time diagnostic purposes.

Another technique of denoising, the wavelet denoising, was used in order to obtain a better detection and to optimize the real-time analysis. The simultaneous use of wavelet denoising and SAECG, in postacquisition processing, yielded good results also with short-term ECG. However, the most innovative feature of the work regards the search for parameters, in the time-frequency domain, that confer robustness to the method, as the introduction of a parameter not influenced from the J point location (EN_END_). Finally, a bivariable separation between the time-frequency parameters improved the effectiveness of the method.

Although VLPs can be of great importance in the arrhythmic risk prevention, their detection is not yet widespread, due to the lack of suitable equipment. Our work is an attempt to solve this problem by providing a robust and reliable algorithm.

For clarity, we divided the algorithm into two phases: the preprocessing and the detection phase (Section 2). At the end of Section 2 it will be shown how to adapt the algorithm for the real-time analysis, and in Section 3 we show our results. Finally, in Section 4, the conclusions and future developments will be outlined. 

## 2. Methods

### 2.1. ECG Signals Source

Real electrocardiographic signals, provided by “PhysioNet” database, were used to develop the algorithm. We chose “PTB Diagnostic ECG Database,” a collection of real ECGs acquired by the Physikalisch Technische Bundesanstalt (PTB), the German national metrology institute [2]. The signals are characterized by sampling frequency of 1 kHz, resolution of 16 bit with 0.5 *μ*V/LSB, and total duration of about 2 minutes. Each ECG is made by 15 leads: 12 conventional and 3 orthogonal (Frank leads).

### 2.2. Preprocessing

**Figure 2:**
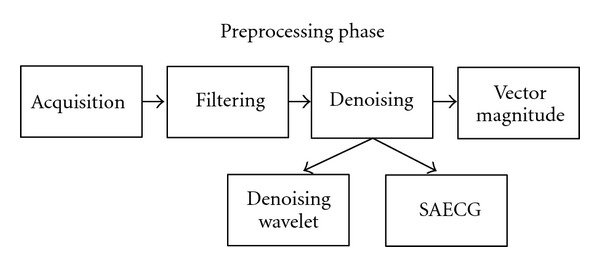
Steps of the pre-processing phase.

The aim of the signal pre-processing is the reduction of the noise level in the ECG record. For this purpose two denoising techniques will be used: the wavelet denoising and SAECG. The end result will be the vector magnitude (VM), which contains the information of all leads in a single signal.  Figure 2 shows the main pre-processing steps.

#### 2.2.1. Filtering

The first operation consists in filtering the signal. The filter performs two functions simultaneously: the first is to remove both the DC component and the low-frequency oscillations (which can be due for example to breathing) and the second is to limit the bandwidth to the component of interest, in order to limit the noise energy.

**Figure 3:**
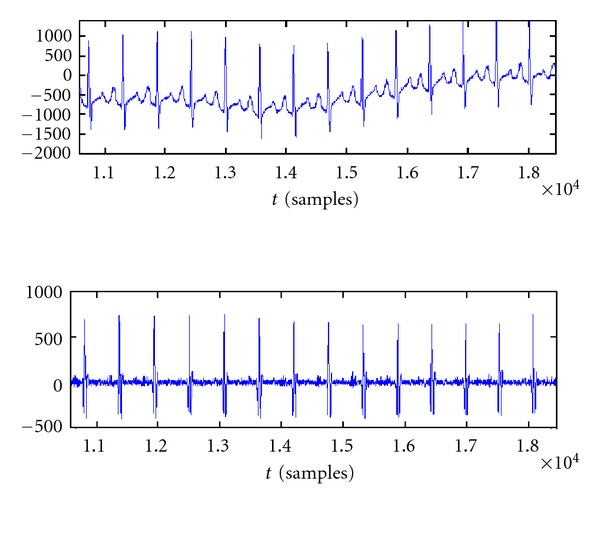
Effect of a fourth-order Butterworth filter on an ECG signal.

The studies carried out by Jane Raimon, Pablo Laguna, and Pere Caminal have demonstrated the superiority of nonlinear phase filters for this application. In particular the filter which gives the best results is a band-pass Butterworth filter of the fourth order, with cut-off frequencies at 25 Hz and 300 Hz.  Figure 3 shows its effect on an ECG signal [3].

**Figure 4:**
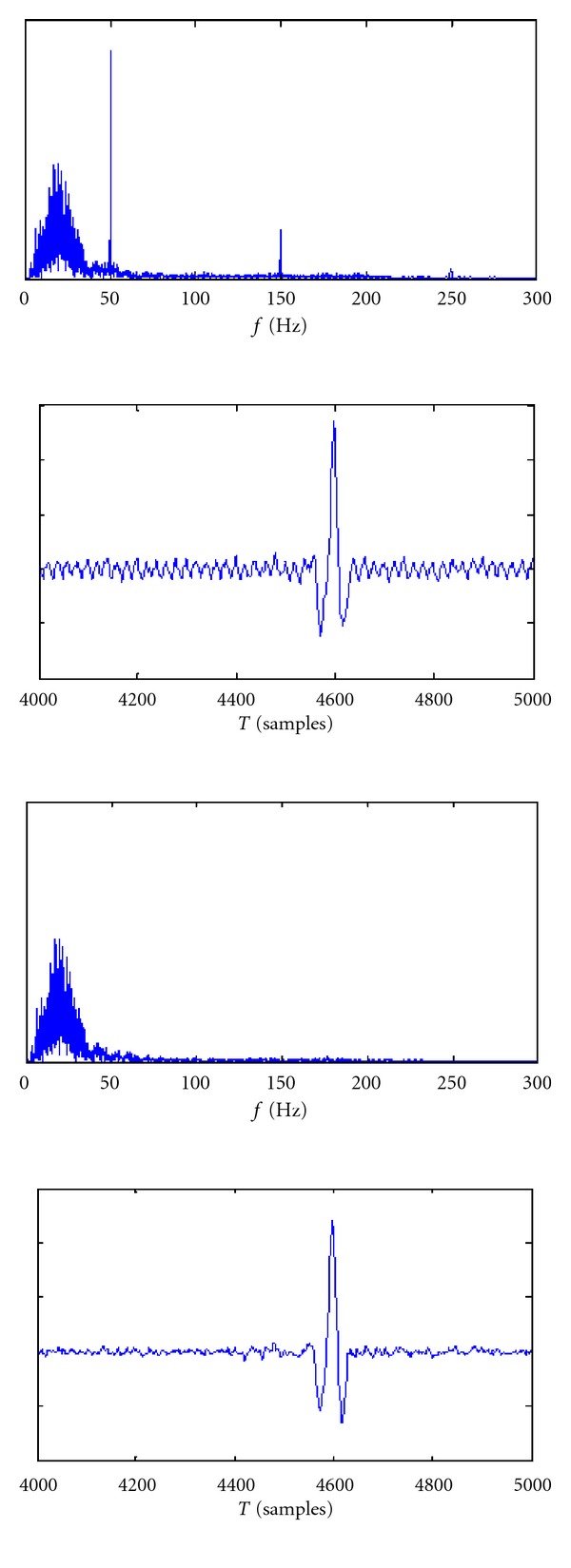
Effect of a second-order Butterworth notch filter on ECG and its frequency spectrum.

In some signals we found the presence of peaks in frequency, located at 50 Hz and its harmonics, which are most likely due to a poor filtering of the power supply in the acquisition phase. So it was necessary to remove these components with notch filters. We used a second-order filter, with attenuated band of 5 Hz around the detected peak. The effect of the filter is shown in Figure 4.

This operation can be performed either in the acquisition phase or after the calculation of the SAECG.

#### 2.2.2. SAECG

One of the first methods developed to reduce the noise power in an electrocardiographic signal is the signal averaged electrocardiography (SAECG). This method is highly effective, and it is used in the preliminary analysis of all algorithms for VLPs detection. It is based on the principle according to which, averaging *N* realizations of a Gaussian stationary process, its variance is reduced by a factor *N*. The individual beats of an ECG can be considered as embodiments of a process having an aleatory component with the above-mentioned characteristics, due to noise, and a deterministic component, the useful signal.

The SAECG is, therefore, obtained by averaging each of these individual beats, to obtain a signal with a very low noise level, suitable for the detection of small-intensity signals, as the VLP. The voltage noise, however, is proportional to the standard deviation, which is reduced by a factor $1/\sqrt{N}$, instead of 1/*N* as the variance. Therefore, the noise level is not reduced proportionally to the number of beats considered. One of the main problems of SAECG is the exact alignment of beats, to leave unchanged the deterministic component; even a slight displacement can make the method ineffective.

Several techniques have been developed for the beats alignment. In particular the authors Jane et al. have distinguished three main variants that include all the methods used in VLP detection algorithms [4].

**Figure 5:**
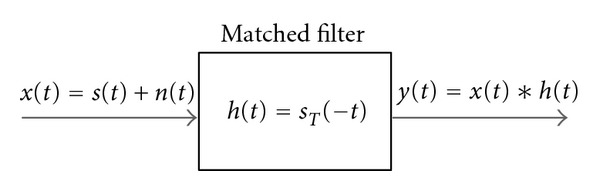
LTI system of a matched filter.

(a) Double-level method: an amplitude threshold is set in such a way that the signal exceeds it only in correspondence of the QRS complexes. The alignment is determined averaging the instant at which the threshold is exceeded by the signal and the instant in which the level returns below it.
(b) Normalized integrals method: each beat, called *v*(*t*), is considered as a scaled and translated copy of a template beat *s*(*t*) (1):
  (1) $$\begin{array}{c} v\left(t\right)=ks\left(t-d\right), \end{array}$$
  where “*k*” is a constant and “*d*” is the delay between *v*(*t*) and *s*(*t*). The delay between the beat and the template is determined by comparing their normalized integrals, respectively, *S*(*t*) and *V*(*t*), defined as in
  (2) $$\begin{array}{c} S\left(t\right)=\frac{1}{A}\int_{-\infty }^{t}s\left(\tau \right)d\tau \;\text{where}\;\;A=\int_{-\infty }^{+\infty }s\left(t\right)dt. \end{array}$$
  The delay “*d*”, for which it is possible to achieve the alignment, is obtained from
  (3) $$\begin{array}{c} d=\int_{-\infty }^{+\infty }\left(S\left(t\right)-V\left(t\right)\right)dt. \end{array}$$
(c) Matched filtering method: this is a classical method for the detection of a known signal in the presence of additive white Gaussian noise. It consists of a linear time-invariant system with impulse response *s*(−*t*). If we consider the electrocardiographic signal *x*(*t*) as a sum of a deterministic signal *s*(*t*) and a noise component *n*(*t*), we obtain the system in Figure 5.The impulse response is referred to as a template beat *s*_*T*_(*t*). On the output *y*(*t*) there will be peaks, easily localizable, corresponding to the perfect alignment instants.

**Figure 6:**
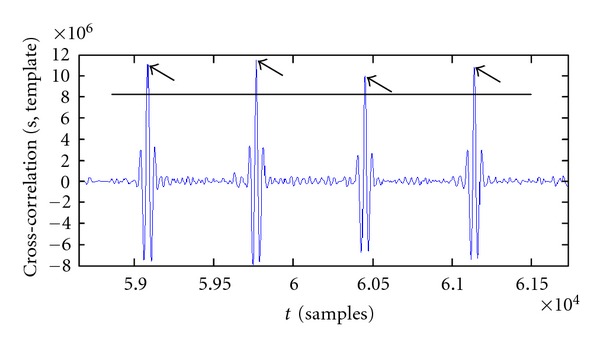
Cross-correlation between template and signal. The arrows show the peaks to be considered for the alignment.

The latter is the most recent and effective method. In the algorithm we use a variant of it, introduced by CD Woody [4], which use the cross-correlation between the beat and the template signal. In the output one gets the same peaks in correspondence of alignment instants, and the principle of the method remains the same. The result of this operation is shown in Figure 6.

The relative maximum of the cross-correlation, marked in Figure 6 and easily determinable, identifies the time shift to be applied in order to optimally align the template. It is unnecessary to repeat the operation for each lead; the reasonable choice is to consider the lead with the greatest amplitude, having the best resolution, and to use the same instants for all the others.

**Figure 7:**
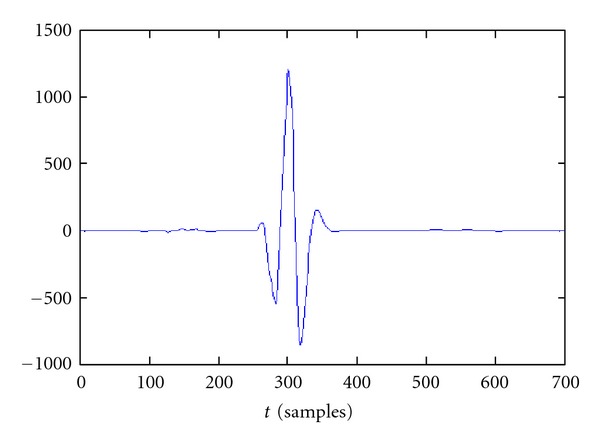
Example of SAECG.

The SAECG is obtained by aligning the individual beats and averaging them, repeating this process for each lead available (Figure 7).

#### 2.2.3. Wavelet Denoising

**Figure 8:**
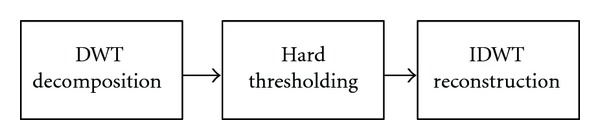
Wavelet denoising general scheme.

The effectiveness of the SAECG increases with the number of beats analyzed. However, it is not always possible to operate on long time ECG; in these cases it is therefore necessary to use alternative denoising methods. For this purpose we have chosen, for its direct effect on individual beats, the wavelet denoising. Details of this technique can be found in [5]. In Figure 8, the main steps of wavelet denoising are shown, using Discrete Wavelet Transform (DWT) and inverse discrete wavelet transform (IDWT).

 For decomposition and reconstruction we used the family of wavelets “Coiflets 5,” which gives the best results, decomposing the signal into five levels. The choice of the threshold value is very important, we do not recommend to set this value as a constant. Better results are obtained by setting a threshold proportional to the residual noise. The noise level is measured in the second level of decomposition, calculating the standard deviation of the signal in a section in which there is only noise. *Hard thresholding* was used to cancel the coefficients below the threshold [6].

#### 2.2.4. Vector Magnitude (VM)

The vector magnitude quantifies the energy measured by the three bipolar leads:

(4) $$\begin{array}{c} \text{VM}=\sqrt{X^{2}\left(t\right)+Y^{2}\left(t\right)+Z^{2}\left(t\right)}, \end{array}$$

where *X*(*t*), *Y*(*t*), and *Z*(*t*) are the SAECG of the three leads. Therefore, the VM contains the information related to all the considered leads. We propose instead to extend its definition to all available leads:

(5) $$\begin{array}{c} \text{V}{\text{M}}_{\text{TOT}}=\sqrt{\sum_{n=1}^{N}\text{SAEC}{\text{G}}_{n}^{2}}, \end{array}$$

where SAECG_*n*_ is the SAECG of the *n*th lead and *N* is the total number of available leads.

**Figure 9:**
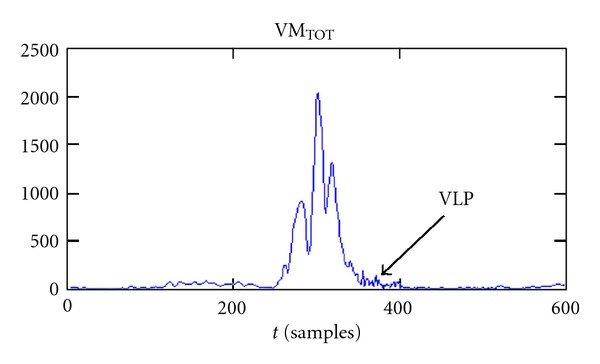
Example of a VM relative to an ECG with VLPs.

The result of the pre-processing phase is shown in Figure 9.

### 2.3. VLP Detection

**Figure 10:**
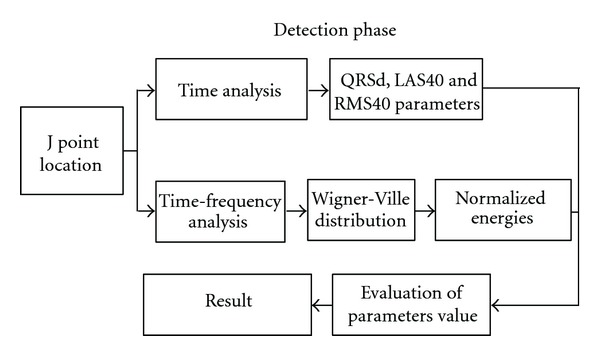
Steps of the detection phase.

The aim of this phase is the measurement of parameters that are directly influenced by the presence of VLPs. To make a comparison we performed both time and time-frequency analysis.  Figure 10 summarizes the main steps of the detection phase. In particular, we compare two techniques, one based on the time analyses and the other based on time-frequency analysis.

#### 2.3.1. J Point Location

The J point marks the end of the QRS complex. In a healthy ECG, it separates a section characterized by a wide signal from a section without electrical activity. In the presence of VLPs this separation is not so sharp, and it is difficult to locate the point. We propose a method for J point location based on energy comparison. The principle was introduced by Legarreta et al. [7].

**Figure 11:**
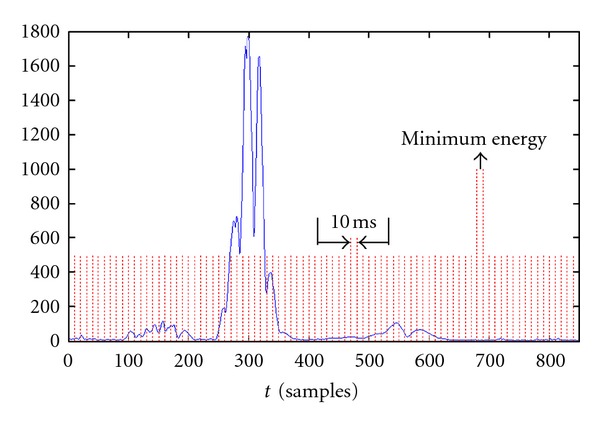
VM division for residual noise estimation.

The first step is the estimation of residual noise level. For this purpose the VM is binned with time steps of 10 msec. Then the energy in each trait is evaluated, and the minimum energy is associated with the noise energy (Figure 11).

An energy threshold, proportional to the noise energy, is then set. Starting from the R wave peak, which is the absolute maximum of the VM, the threshold is compared with the energy evaluated in a 10 ms interval after each ECG sample. The first point that provides an energy below the threshold is considered as the J point.

The same method is then used to locate another reference point on the ECG, we called it “QRSoff.” It represents the end of the QRS complex and has to be independent of the possible presence of VLPs. This means that the energy threshold used for its identification must be significantly greater than the one used for J point detection. This point is useful in time-frequency analysis in case of incorrect location of the J point. The best results were obtained with thresholds about 5 and 100 times greater than the noise energy, respectively, for the J point and QRSoff. However, the optimal values are different by changing the acquisition hardware, and then the signals characteristics. It is, therefore, necessary to test and analyze the results of a substantial number of acquired ECGs, until obtaining a threshold level that allows optimal localization of the points in all signals.

**Figure 12:**
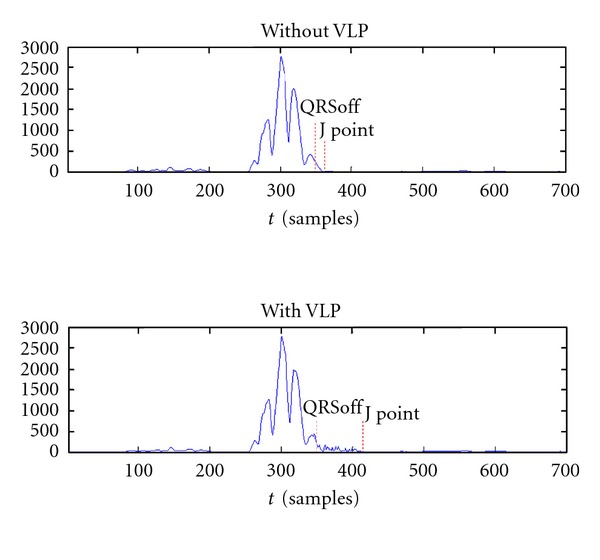
Example of J point and QRSoff location on VM with and without VLP.

Figure 12 shows the points in a VM with and without VLP.

#### 2.3.2. Time Analysis

**Figure 13:**
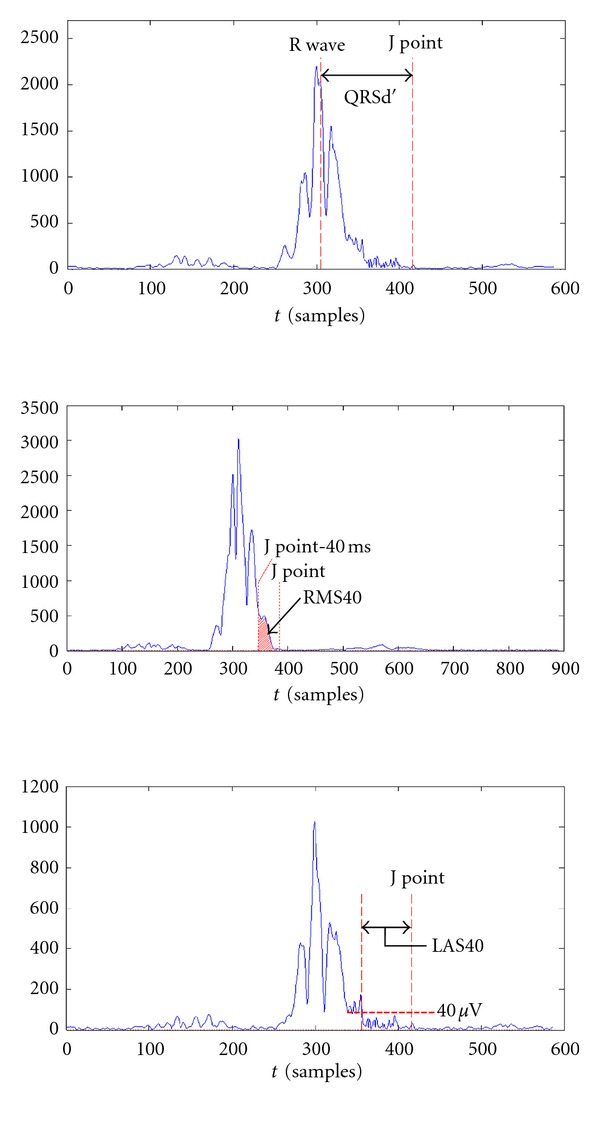
Definition of QRSd (top), RMS40 (middle), and LAS40 (bottom).

Once the J point has been determined, the measurement of temporal parameters is almost immediate. The standard parameters for the time analysis [8] are as follows (Figure 13).

- QRSd: QRS complex duration.
- RMS40: root mean square voltage of the terminal 40 msec of the QRS complex.
- LAS40: amount of time that the QRS complex remains below 40 *μ*V.

The measurement of QRSd requires the knowledge of the QRS complex onset. However, the VLPs are present only at the end of the complex. For this reason we have redefined the parameter QRSd as the temporal distance between the R wave peak and J point, influenced equally by the VLP (Figure 13). Standard analysis is still possible by determining the onset of the QRS complex using the same method introduced for the J point location.

#### 2.3.3. Time-Frequency Analysis

The time-frequency representations (TFRs) are a very effective tool for VLPs detection, due to the localization of VLPs in both domains. Unlike the time analysis, a standard for this approach has not yet been defined. Laciar and Orosco proposed the use of normalized energies as parameters for VLP detection [9]. In their work, they made a comparison between three different representations: the short-time fourier transformer (STFT), Wigner-Ville (WVD) and Choi-Williams (CWD) distributions. They obtained the best results with the WVD, defined in (6), which is what we decided to use in our work:

(6) $$\begin{array}{c} \text{WVD}\left(t,f\right)=\int_{-\infty }^{+\infty }x\left(t+\frac{\tau }{2}\right)\;x^{*}\left(t-\frac{\tau }{2}\right)\;e^{-j2\pi f\tau }d\tau . \end{array}$$

**Figure 14:**
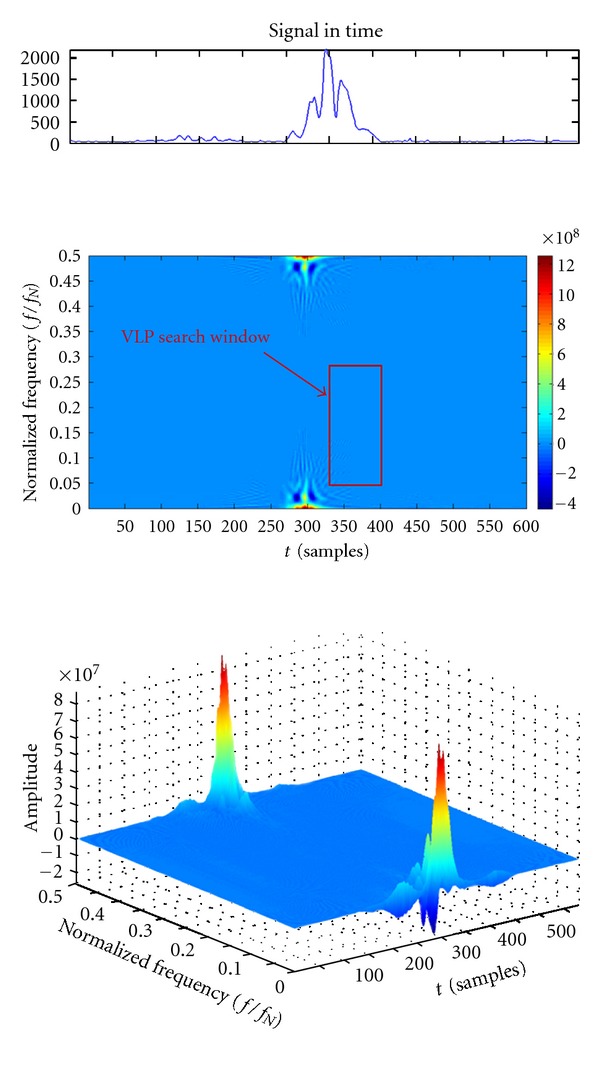
Representation of the VM signal (top) and its WVD (bottom) in the time-frequency plane and in 3D.

Figure 14 shows an example of WVD of an ECG. The window where the VLP should be located is also highlighted.

The normalized frequency in Figure 14 is the frequency divided by the Nyquist frequency *f*_*N*_ (i.e., half the sampling frequency). Moreover, the second peak around *f*/*f*_*N*_ = 0.5 is due to the alias effect.

The first value of energy is calculated in an interval around the J point. The extremes of the window that gave the best results, based on the characteristics of VLP signal, are as follows:

(7) tmin⁡=J  point−55 msec,tmax⁡=J  point+25 msec,fmin⁡=55 Hz,fmax⁡=300 Hz.

 The energy in this area, called *E*_VLP_, is therefore evaluated as in

(8) $$\begin{array}{c} E_{\text{VLP}}=\sqrt{\frac{1}{k_{1}}\sum_{f=f_{\min}}^{f_{\max}}\sum_{t=t_{\min}}^{t_{\max}}{\text{TFR}}^{2}\left(t,f\right)}, \end{array}$$

where TFR is the two-dimensional matrix resulting from the WVD of the signal and *k*_1_ (as *k*_2_ and *k*_3_ in (9)-(10)) is a normalization parameter defined as the product between the rows and columns numbers of the considered region in TFR matrix.

We introduced a second energy value, called *E*_END_, calculated in an area next to the QRSoff point, previously determined (9). 80 msec is a time sufficiently high to analyze the region in which to search VLPs:

(9) $$\begin{array}{c} E_{\text{END}}=\sqrt{\frac{1}{k_{2}}\sum_{f=f_{\min}}^{f_{\max}}\sum_{t=\text{QRSoff}}^{\text{QRSoff}+80\;\text{msec}}{\text{TFR}}^{2}\left(t,f\right)}. \end{array}$$

This value is very important in case of wrong localization of the J point. However, the calculated energies require a normalization. For this purpose, they are divided by the energy of the QRS complex (*E*_QRS_), evaluated from the R wave to the QRSoff:

(10) $$\begin{array}{c} E_{\text{QRS}}=\sqrt{\frac{1}{k_{3}}\sum_{f=f_{\min}}^{f_{\max}}\sum_{t=\text{R wave}}^{\text{QRSoff}}{\text{TFR}}^{2}\left(t,f\right)}. \end{array}$$

The normalized energy indexes are, therefore, as in (11).

(11) $$\begin{array}{c} \text{EN}=\frac{E_{\text{VLP}}}{E_{\text{QRS}}},\\ {\text{EN}}_{\text{END}}=\frac{E_{\text{END}}}{E_{\text{QRS}}}. \end{array}$$

The EN index is lower in patients with VLPs, because, in the neighborhood of the J point, there is less energy compared to a healthy ECG. On the contrary, the presence of VLPs makes the EN_END_ index greater.

### 2.4. Real-Time Processing

Electrophysiological abnormalities may change on a beat-to-beat basis, resulting in a failure of signal-averaged recordings to identify changes related to arrhythmogenesis. The VLP detection during the ECG acquisition is therefore very important. An adequate noise reduction is required in order to obtain a good effectiveness. The impossibility of using the SAECG to increase the signal-noise ratio is the main problem of real-time analysis.

**Figure 15:**
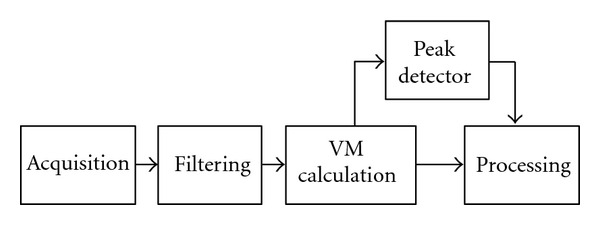
Input stage steps for real-time processing.

The real-time application of the algorithm requires the modification of the input phase. In particular, a peak detector should be introduced in order to identify the individual beats exploiting the high amplitude of the QRS complex. Each heartbeat is then sent to the processing phase. We propose a scheme as in Figure 15.

The processing phase is the same used in the postacquisition analysis, without the SAECG calculation step.

## 3. Results

To test the method we used 60 real electrocardiographic signals with artificial VLP (aVLP). The VLP component has been simulated with a sum of sine signals with a definite amplitude and frequency, added to the first milliseconds of the ST segment [10]:

(12) $$\begin{array}{c} \text{aVLP}\;\left[n\right]=\sum_{i=1}^{N}A_{i}\cos(2\pi f_{i}n). \end{array}$$

The constant *A*_*i*_ is set equal to one hundredth the amplitude of the ECG in the postacquisition analysis, and it assumes different values in the real-time analysis. We have considered a VLP duration of 40 ms and frequencies of 70, 130, 210 and 280 Hz to reproduce realistic VLP.

### 3.1. Postacquisition Analysis

**Figure 16:**
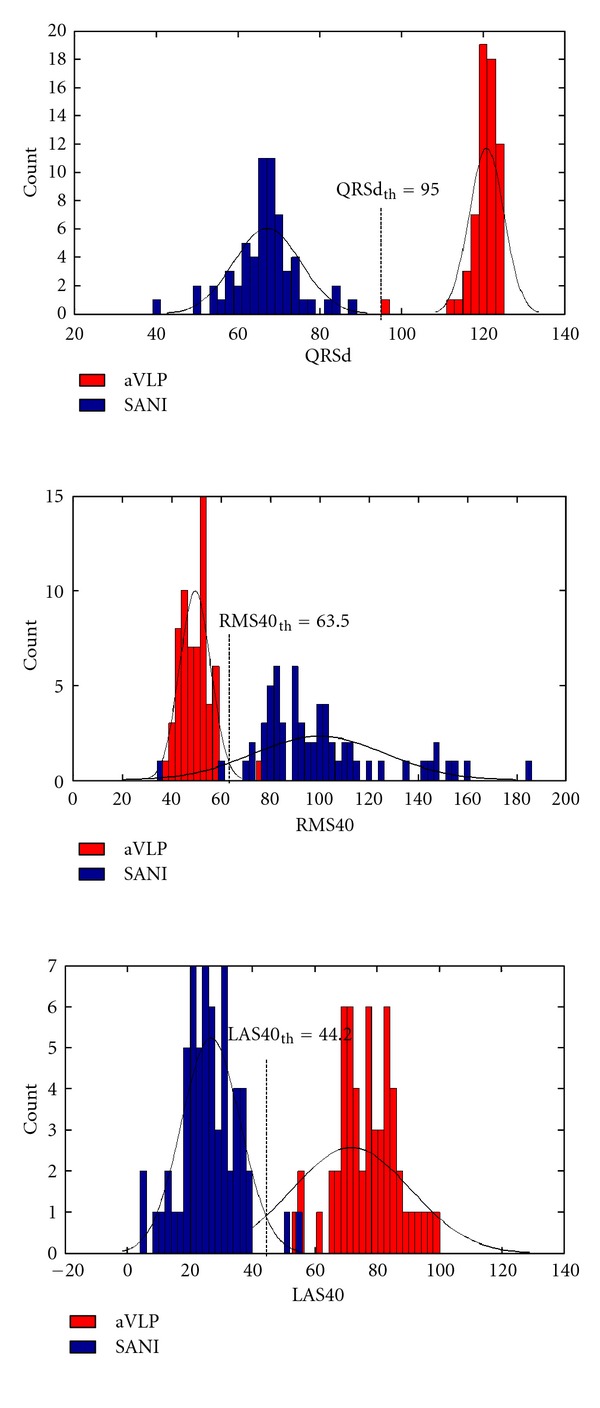
QRSd (top), RMS40 (middle), and LAS40 (bottom) parameters related to healthy ECG (blue) and ECG with aVLP (red).

The results obtained can be displayed by histograms.  Figure 16 shows how the temporal parameters divide healthy ECG from the group with aVLP.

The parameters are distributed according to a Gaussian law. Our optimal threshold values, as well as the corresponding effectiveness, are reported in Table 2.

**Figure 17:**
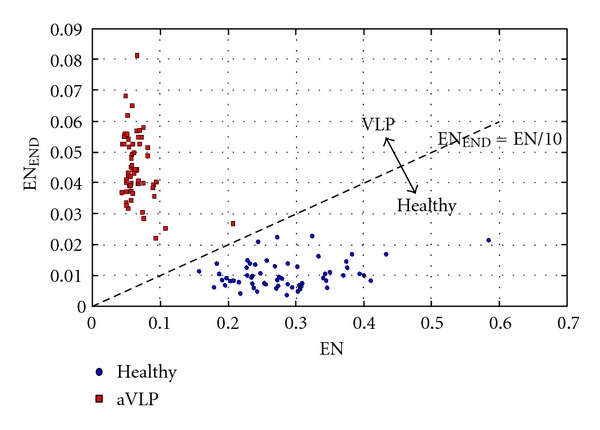
Bivariable time-frequency parameters separation between healthy ECG (blue) and ECG with aVLP (red).

For time-frequency parameters we chose to use bivariable analysis, to improve the effectiveness [11].  Figure 17 shows the separation between the healthy ECG and ECG with aVLP.

Using the straight line EN_END_ = EN/10 we obtained a complete separation between the two groups. This separation is not unique but heavily depends on acquisition hardware and signals characteristics.

### 3.2. Real-Time Analysis

**Figure 18:**
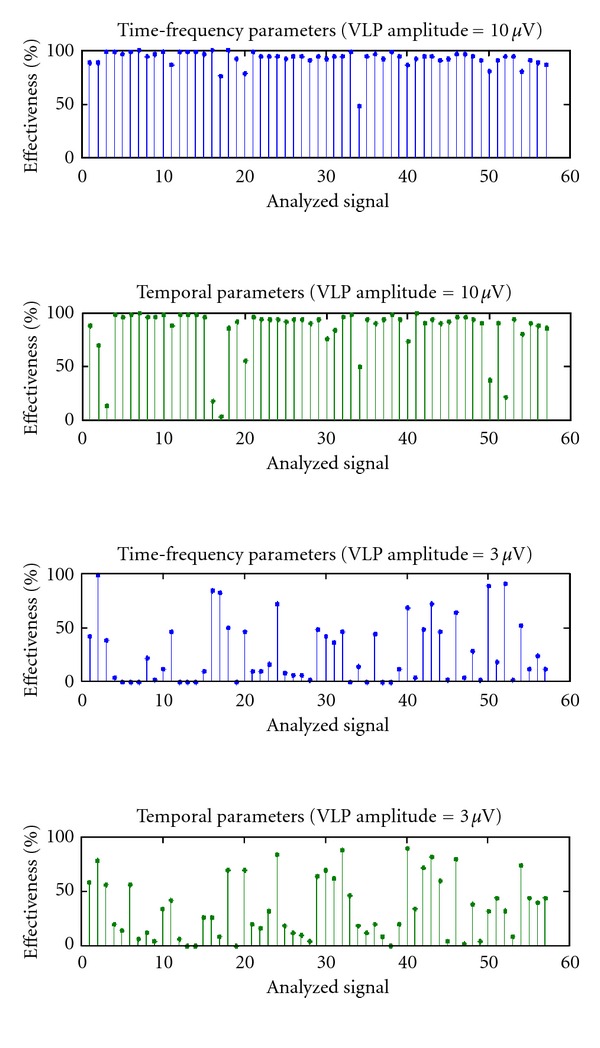
Values of effectiveness for each analyzed signal, with both time-frequency analysis (graphic at the top) and temporal analysis (graphic in the lower). VLP amplitude of 10 *μ*V (top figure) and 3 *μ*V (bottom figure).

To test the effectiveness of the real-time processing we have generated signals with a known number of aVLPs. The effectiveness represents the ratio between the number of VLPs detected and the real number of VLPs in the signal. For the temporal analysis the presence of VLPs was confirmed if at least two of the three parameters (QRSd, LAS40, and RMS40) were outside the established range. Instead, for the time-frequency analysis, VLPs were confirmed if the point in the plane (EN, EN_END_) exceeds the separation line EN_END_ = EN/10.  Figure 18 shows the effectiveness obtained on each analyzed signal with aVLP, respectively, by 10 and 3 *μ*V.

**Figure 19:**
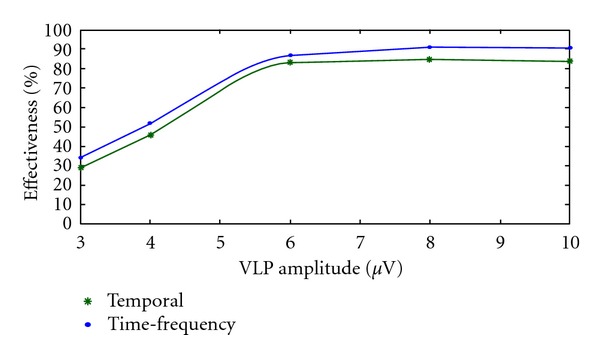
Average effectiveness obtained with VLPs of different amplitude (3, 4, 6, 8, and 10 *μ*V). Comparison between temporal analysis (green line) and time-frequency analysis (blue line).

It is clear that 3 *μ*V VLPs are not detectable. The graph in Figure 19 was obtained by plotting the average effectiveness related to VLPs of different amplitude.

## 4. Discussion

We developed a new method for ventricular late potentials (VLP) detection in high-resolution electrocardiographic (HRECG) signals, suitable for both postacquisition analysis and real-time applications. The method was evaluated with a group of 60 healthy ECG, provided by “PTB Diagnostic ECGs Database,” with the addition of realistic artificial VLP (aVLP).

The innovative features of the work, in addition to taking advantages of the best methods available in the literature, concerning a phase of preprocessing with the simultaneous use of two denoising techniques (the SAECG and the denoising wavelet) and a detection phase based on the bivariable separation of time-frequency parameters. Very important is the independence of the parameter EN_END_ from the J point location, which may be easily subject to wrong localization.

A comparison between the time and the time-frequency analysis demonstrated the real improvement achievable with the time-frequency parameters. The results obtained show how, with the time-frequency representation, it is possible to have an effectiveness of 100%, using the signal averaged electrocardiography (SAECG), and an effectiveness higher than 90% for the beat-to-beat analysis, considering VLP of amplitude greater than 6 *μ*V. Instead, the temporal parameters QRSd, RMS40, and LAS40 divide the two groups of ECG with an effectiveness, respectively, of 96.7%, 91.7%, and 85%. It is also possible to further improve the results using high-performance acquisition hardware and low-noise electrodes.

The algorithm for automatic VLP detection can be implemented by software, for example in the Holter ECG, or by firmware, directly associated with an acquisition hardware. Future developments may include a statistical study of a large number of ECGs in order to standardize the thresholds of parameters and optimize the time-frequency analysis.

## Figures and Tables

**Table 1:** *Characteristics of VLPs*.

Ventricular late potentials (VLPs)	
Causes	Areas of myocardium with reduced conductivity
Effects	Onset of serious arrhythmias
Bandwidth	40–300 Hz
Time location	End of QRS complex and initial part of ST segment
Duration	<50 msec
Amplitude	1–20 *μ*V

**Table 2:** Effectiveness of temporal parameters.

Temporal parameters		
Parameter	Threshold	Effectiveness
QRSd	95	96.7%
RMS40	63.5	91.7%
LAS40	44.2	85%
